# Legacy of the GDR: regional disparities in childhood maltreatment in post-unification Germany

**DOI:** 10.1186/s13034-025-00876-7

**Published:** 2025-03-20

**Authors:** C. Kasinger, S. Heiner, A. Heller, T. McLaren, M. Beutel, V. Clemens, E. Brähler

**Affiliations:** 1 Department for Psychosomatic Medicine and Psychotherapy, University Medical Center of Johannes Gutenberg University of Mainz, Untere Zahlbacher Str. 8, 55131 Mainz, Germany; 2 GESIS - Leibniz-Institute for the Social Sciences, Mannheim, Germany; 3 Department of Psychiatry and Psychotherapy, Leipzig University Hospital, Leipzig, Germany; 4 Department for Child and Adolescent Psychiatry/Psychotherapy, University of Ulm, Ulm, Germany; 5 Department of Medical Psychology and Medical Sociology, University of Leipzig, Leipzig, Germany

**Keywords:** Childhood Maltreatment, Germany, Transgenerational, Unification, Abuse, Neglect

## Abstract

**Background:**

Previous research has identified growing up in the former German Democratic Republic (GDR) as a protective factor against childhood abuse and neglect compared to growing up in the former Federal Republic of Germany (FRG). This study investigates whether these differences persist among individuals born after German reunification, providing a novel focus on the post-unification generation and the possible transgenerational transmission of childhood maltreatment in Germany.

**Method:**

The prevalence of self-reported abusive and neglected childhood experiences among 17- to 31-year-olds, stratified by gender and socio-political context (East/West), was examined using the Childhood Trauma Questionnaire (CTQ). Data of the German Health Interview and Examination Survey for Children and Adolescents (KIGGS-study) between 2014 and 2017 was used. Logistic regression models were used to assess the influence of covariates such as age, subjective social status, and education on childhood trauma experiences.

**Results:**

Altogether, 18.6% (East = 15.8%/ West = 19.0%) of the *N* = 5,982 participants reported at least one type of maltreatment. The prevalence of at least moderate abuse was found to be 6.8% (East = 5.8%/West = 7.0%) for emotional, 3.7% (East = 2.4%/West = 3.9%) for physical, and 3.3% (East = 1.9%/West = 3.6%) for sexual abuse, and a prevalence of 9.2% (East = 8.5%/West = 9.3%) for emotional and 8.7% (East = 7.4%/West = 8.9%) for physical neglect. Individuals residing in eastern German regions reported significant lower prevalence rates for sexual and physical abuse, as well as overall maltreatment.

**Conclusion:**

Significant regional disparities in childhood maltreatment were observed among individuals raised in post-unification Germany, suggesting the persistence of sociopolitical influences from the former GDR as a protective factor. These findings underscore the importance of understanding transgenerational transmission factors of childhood maltreatment, such as parenting behaviors within differing sociopolitical contexts. The results have important implications for child welfare policies, emphasizing the need to address regional disparities and to incorporate historical and sociopolitical factors into future research and intervention strategies.

**Supplementary Information:**

The online version contains supplementary material available at 10.1186/s13034-025-00876-7.

## Introduction

Experiences of diverse forms of violence or neglect during childhood and adolescence can lead to significant psychological consequences that persist into adulthood. Children are particularly vulnerable to adverse experiences due to their dependence on caregivers and the incomplete maturation of their cognitive and emotional processes [1]. These experiences are associated with an elevated risk of psychopathological conditions such as depression [2, 3], anxiety disorders, and substance abuse [4, 5]. They are also linked to behavioral challenges, including aggressive tendencies and interpersonal difficulties [6]. In addition, a meta-analysis conducted by Wegman and Stetler [7] revealed a correlation between childhood experiences of abuse and neglect and an overall poorer physical health status in adulthood. In Germany, the prevalence rates for at least moderate maltreatment are estimated to be between 1.6 and 2.8% for abuse and 6.6–10.8% for neglect [8, 9]. A previous analysis of the same dataset reported the following prevalence rates: 6.7% reported experiences of emotional abuse, 3.7% physical abuse, 3.5% sexual abuse, 9.0% emotional neglect and 8.6% physical neglect. Overall, 18.4% of the participants reported having experienced at least one type of CM [10]. Notably, a gender-specific pattern emerged, with female participants exhibiting a consistently higher risk of experiencing sexual and emotional abuse.

Given the profound developmental implications of child abuse and neglect, it is of paramount importance to enhance our understanding of protective and risk factors in order to identify potential hazards and advance the development of effective prevention and intervention strategies. Familial risk factors such as parental mental illness, substance abuse, domestic violence, lower parental education, poverty, and unemployment play a significant role [11–14]. Child-specific characteristics, including gender, also play a pivotal role [15]. Furthermore, there are also municipal risk factors, including deprived and geographically isolated neighborhoods [16]. From a broader sociopolitical perspective, economic conditions [17, 18], gender equality [19] and legal frameworks, such as laws prohibiting corporal punishment [20], have been identified as important factors, that influence the prevalence rates of childhood maltreatment.

In light of the aforementioned considerations, regarding municipal and sociopolitical risk and protective factors, the German division and unification present a singular opportunity to examine the impact of disparate sociopolitical contexts. Previous studies have indicated that instances of child maltreatment and neglect are less frequently reported in eastern than in western German states [21, 22]. This is attributed, at least in part, to the sociopolitical context of the German Democratic Republic (GDR), where corporal punishment was prohibited from an early stage, and the option of early childcare outside the family was common and could have relieved pressure on families and contributed to greater financial independence for women [21]. However, prior research has largely focused on individuals who directly experienced the GDR era, leaving a critical gap in understanding the persistence of these effects among the “post-unification generation”—individuals raised in unified Germany but socialized within the legacies of eastern or western sociopolitical contexts.

Research repeatedly shows that individuals who experienced abusive and neglectful childhood experiences are more likely to afflict abusive and neglectful childhood experiences on their children as well [23]. Theoretical frameworks on this transgenerational transmission of child abuse and neglect have largely focused on transmission mechanisms located in the parenting individuals, such as mental health and emotion regulation issues and a disturbed biological stress response due to traumatic childhood experiences. Additionally, potential genetic transmission mechanisms, such as genetic predisposition and epigenetic embedding and inheritance of maltreatment are widely discussed [24]. Apart from factors like poverty and structural disadvantages there is little emphasis on how sociopolitical environments shape parenting practices across generations. Parenting practices may be also influenced by caregiving strategies, such as the acceptance of corporal punishment and the power gradient between the sexes in a society. These factors are at work through generations and are widely shaped by broader societal norms, which may explain regional disparities in child-rearing practices and childhood maltreatment. In the case of Germany for instance individuals, who grew up in the former GDR remember the parenting of their parents as warmer and less harsh than individuals from the former Federal Republic of Germany (FDR) [25].

This study seeks to address these gaps by presenting the first nationwide data on the prevalence of childhood maltreatment among individuals who have grown up in the unified Germany. We hypothesize that the sociopolitical legacies of the GDR will persist through transgenerational mechanisms, resulting in lower rates of childhood maltreatment in eastern Germany compared to western Germany among the post-unification generation.

## Methods

### Participants

The data set was derived from a representative survey conducted by the Robert Koch-Institut as part of the German Health Interview and Examination Survey for Children and Adolescents (KiGGS). The participants were selected via a two-stage random sampling process, with the assistance of the local population registries. The study comprises a baseline survey and two follow-up surveys, enabling cross-sectional and longitudinal analyses. The data utilized in this study were derived from the second follow-up survey (KiGGS Wave 2), which was conducted via written and postal questionnaires between 2014 and 2017. Of the original sample of 17,641 individuals surveyed at the baseline, *N =* 10,853 were successfully re-contacted, representing a response rate of 61.5%. Individuals residing in Berlin were excluded from the study (*n* = 409), as it was not feasible to assign them to the former eastern or western German states. However, for reasons of transparency and to provide additional context the prevalence rates for childhood maltreatment in Berlin are presented in Table 4 in the supplement materials. To ensure the representativeness of the data, a weighting factor provided by the authors of the KiGGS study was applied to correct for sample deviations. This weighting factor adjusts the sample to align with the official population statistics as of December 31, 2015, considering variables such as age, gender, federal state, nationality, and parental educational attainment [26]. Additionally, it compensates for panel attrition effects, as the KiGGS survey tends to under-represent participants from lower socioeconomic backgrounds, older age groups, and individuals with immigrant backgrounds [27]. Only those participants who achieved a total score on at least one of the Childhood Trauma Questionnaire (CTQ) subscales were included in the analyses. Furthermore, only individuals who had reached the age of 18 years or older were included in the study, as only those individuals were presented with the CTQ. This resulted in a total sample of *N* = 5,982 (mean age = 22.75, age range = 18 to 31 years, birth years: 1985–1999, female = 48.3%). The study was reviewed and approved by the ethics committee of the Medical University of Hannover (reference number 2275 − 2014). Participants were informed in detail about the study, the voluntary nature of participation, and data protection, and provided written consent.

### Material

#### Childhood experiences of abuse and neglect

The internationally established Childhood Trauma Questionnaire (CTQ) [28–31] comprises 28 items for retrospective assessment of maltreatment and neglect during childhood and adolescence. The questionnaire comprises five subdimensions, each with five items. These are: physical and emotional neglect, physical and emotional abuse, and sexual abuse. The internal consistency of the subdimensions was acceptable to good, with McDonald’s Omega coefficients of 0.826 for emotional maltreatment, 0.769 for physical abuse, 0.943 for sexual abuse, and 0.886 for emotional neglect. The only subscale to demonstrate poor internal consistency was pertaining to physical neglect (ω = 0.365).

The individual items of each CTQ dimension were aggregated into a total score upon completion of the questionnaire. For each subscale severity scores were calculated based on norm data provided by Haeuser et al. [9]. The categories for the severity scores are “none-minimal”, “minimal-moderate”, “moderate-severe”, to “severe-extreme”. Dichotomous scores (e.g. experience of physical abuse: yes/no) were based on scores reaching at least the moderate-severe level [8, 9]. The cut-off score for emotional abuse was 13, for physical abuse and neglect 10, and for emotional neglect 15. A score of 8 was used as the cut-off for sexual abuse. The disparate cut-off values are a consequence of the differing traumatic qualities inherent to experiences of maltreatment and neglect and are distinguished in accordance with the approach of Glaesmer et al. [31].

#### Socio-demographic variables

The participants were assigned to the “West” (0) and “East” (1) groups based on their respective federal states of their current place of residence. The variable indicating gender was recorded using a binary system, with male coded as 1 and female as 2. The subjective social status (SSS) was assessed using the German version of the MacArthur Scale, which is represented by a ten-step ladder. Subsequently, the respondents are asked to position themselves on a scale of perceived social status. The highest level is defined as one in which individuals possess the greatest financial resources, educational attainments, and professional positions [32]. Thus, the assessment is based on a subjective comparison with other individuals in Germany. Information regarding education was collected through the inquiry about the highest level of general education attained. For the logistic regression models, a dichotomous variable was created to indicate whether or not an individual had obtained an A-level certificate (equivalent to high school diploma). Comprehensive details about the variables can be found in the frequency table of the sociodemographic information (Table 1).

**Table 1:** Socio-Demographic variables

	All	East	West
	*N* = 5982	*N* = 762 (12.7%)	*N* = 5.220 (87.3%)
**Gender (N / %)**			
Male	3.093 (51.7)	403 (52.9)	2.690 (51.5)
Female	2.889 (48.3)	359 (47.1)	2.530 (48.5)
**Age (MW / SD)**			
Age in Years	22,75 (3,25)	22,68 (3,24)	22,78 (3,25)
**High School diploma**			
Yes	2.830 (47.7)	309 (40.8)	2.521 (48.7)
No	3.105 (52.3)	448 (59.2)	2.657 (51.3)
Missing	47	5	42
**Subjective social status**			
**(MW / SD)**	5.34 (1.67)	5.14 (1.65)	5,37 (1.67)
Missing	201 (3.3)	23 (3,0)	178 (3.4)

MW: Mean; SD: Standard deviation

### Statistics

All analyses were conducted using the statistical software package SPSS (SPSS Statistics 26). The binary variable described in Chap. 2.2, based on the sum score of the respective CTQ subdimensions, was used as the outcome variable for four logistic regressions. The predictor variable was the east-west allocation, while other covariates included gender, age, subjective social status, and a binary variable for educational attainment. To ensure that our findings are not a statistical artefact due to the possible danger of oversimplification of binary coding, we also performed a linear regression on the severity of abuse and neglect. The results of these additional analyses are presented in Table 5 in the supplement materials. To mitigate the potential impact of the sample size disparity between East and West Germans and to ensure the reliability of the findings, we conducted robustness checks, performing additional bootstrapping logistic regression analyses. The results of the bootstrapping analyses, confirmed the same trends observed in the primary analysis, further supporting the robustness of our findings despite the sample size disparity.

## Results

### The presentation of childhood experiences of abuse and neglect

Table 2 depicts the self-reported prevalence rates of abusive and neglected childhood experiences. A total of 6.8% of respondents indicated that they had experienced emotional abuse, while 9.2% reported experiencing emotional neglect and 8.7% reported experiencing physical neglect. Lower percentages are observed on the scales of physical (3.7%) and sexual abuse (3.3%). At least one type of maltreatment experienced 18.6% of the participants. Upon examination of the gender-specific breakdown, it becomes evident that the prevalence rates are higher among girls and young women, particularly in the cases of emotional and sexual abuse. Additionally, the West German sample exhibits higher prevalence rates across all subscales, with the exception of physical neglect, when compared to the East German sample.

**Table 2:** Prevalences of abusive and neglectful childhood experiences of the sub-dimensions. Total, by East-West and gender

	All		East		West		Male		Female	
	*N* = 5,*982*		*N* = 762		*N* = 5,*220*		*N* = 3,*093*		*N* = 2,*889*	
**At least one type of Maltreatment**	***N***	***%***	**N**	***%***	**N**	***%***	**N**	***%***	**N**	***%***
yes	1,080	18.6	117	15.8	963	19.0	484	16.1	598	21.3
no	4,731	81.4	623	84.2	4,108	81.0	2,516	83.9	2,215	78.7
**Emotional Abuse**										*%*
yes	406	*6.8*	44	*5.8*	362	*7.0*	149	*4.9*	257	*8.9*
no	5,528	*93.2*	710	*94.2*	4,818	*93.0*	2,910	*95.1*	2,618	*91.1*
**Physical Abuse**										
yes	219	*3.7*	18	*2.4*	201	*3.9*	102	*3.3*	117	*4.1*
no	5,704	*96.3*	732	*97.6*	4,972	*96.1*	2,960	*96.7*	2,744	*95.9*
**Sexual Abuse**										
yes	197	*3.3*	14	*1.9*	183	*3.6*	47	*1.6*	151	*5.3*
no	5,695	*96.7*	736	*98.1*	4,959	*96.4*	2,984	*98.4*	2,712	*94.7*
**Emotional neglect**										
yes	543	*9.2*	64	*8.5*	479	*9.3*	254	*8.3*	289	*10.1*
no	5,370	*90.8*	689	*91.5*	4,681	*90.7*	2,811	*91.7*	2,560	*89.9*
**Physical neglect**										
yes	519	*8.7*	56	*7.4*	463	*8.9*	266	*8.7*	253	*8.8*
no	5,419	*91.3*	701	*92.6*	4,718	*91.1*	2,801	*91.3*	2,619	*91.2*

### Regression analysis

Table 3 presents the results of the regression analysis. Affiliation with the “West” group was found to be a significant predictor of reported physical abuse and sexual abuse as well as experiencing at least one type of maltreatment. Significant differences were also observed with regard to the predictor variable of gender. The logistic regression suggests that females are at an elevated risk for childhood experiences of abuse and neglect, particularly in the context of sexual and emotional abuse as well as experiencing at least one form of maltreatment in general. Low educational attainment is associated with an elevated risk of physical abuse, sexual abuse, and emotional and physical neglect. A low subjective social status is a significant risk factor for childhood abuse and neglect in all domains. Additionally low educational attainment and low subjective social status were significant predictors for experiencing at least one type of maltreatment. In addition to the binary analyses, linear regression models examining the severity scores of different forms of abuse and neglect revealed similar results: individuals from East Germany reported significantly less severe experiences of all forms of abuse compared to those from West Germany (see Table 5 in the Supplement Materials).

**Table 3:** Results of the logistic regression analysis

Dependent Variable	Independent Variable	Odds Ration (OR)	95% confidence interval (CI)	*p*
**At least one type of maltreatment**	*Gender*	1.377	1.200–1.581	< 0.001***
(*N* = 5,597)	*Age*	1.058	1.036–1.080	< 0.001***
	*East-West*	0.717	0.577 − 0.889	< 0.001***
	*High School diploma/ none*	0.734	0.636 − 0.889	< 0.001***
	*Subjective social status*	0.519	0.448 − 0.600	< 0.001***
**Emotional Abuse**	*Gender*	1.842	1.490–2.277	< 0.001***
(*N* = 5,713)	*Age*	1.039	1.007–1.072	0.017*
	*East-West*	0.730	0.524–1.018	0.063
	*High School diploma/ none*	0.949	0.765–1.177	0.634
	*Subjective social status*	0.399	0.316 − 0.503	< 0.001***
**Physical Abuse**	*Gender*	1.265	0.958–1.667	0.098
(*N* = 5,701)	*Age*	1.045	1.002–1.089	0.038*
	*East-West*	0.481	0.285 − 0.811	0.006**
	*High School diploma/ none*	0.568	0.420 − 0.769	< 0.001***
	*Subjective social status*	0.597	0.442 − 0.806	< 0.001***
**Sexual Abuse**	*Gender*	3.522	2.517–4.928	< 0.001***
(*N* = 5,672)	*Age*	1.091	1.043–1.141	> 0.001***
	*East-West*	0.249	0.249 − 0.771	< 0.01**
	*High School diploma/ none*	0.534	0.534 − 0.989	0.042*
	*Subjective social status*	0.375	0.375 − 0.713	< 0.001***
**Emotional Neglect**	*Gender*	1.257	1.047–1.509	0.014*
(*N* = 5,693)	*Age*	1.048	1.019–1.077	< 0.001***
	*East-West*	0.811	0.614–1.073	0.142
	*High School diploma/ none*	0.539	0.441 − 0.659	< 0.001***
	*Subjective social status*	0.450	0.367 − 0.552	< 0.001***
**Physical Neglect**	*Gender*	1.034	0.859–1.244	0.723
(*N* = 5,719)	*Age*	1.089	1.058–1.120	< 0.001***
	*East-West*	0.730	0.545 − 0.979	0.036*
	*High School diploma/ none*	0.670	0.550 − 0.815	< 0.001***
	*Subjective social status*	0.622	0.511 − 0.758	< 0.001***

ß: beta-weight; significance levels: *** *p* <.001, ** *p* <.01, * *p* <.05

## Discussion

This study provides novel insights into regional differences in CM by focusing on individuals from the post-unification generation, who were not directly exposed to the sociopolitical environment of the GDR. Our findings reveal a lower prevalence of CM among individuals from East Germany compared to their West German counterparts. Specifically, logistic regression models indicated a significantly elevated risk for potentially traumatic childhood experiences in the subdimensions of experiencing at least one type of maltreatment, sexual and physical abuse as well as physical neglect for individuals raised in West Germany. These results add to the growing body of research on regional disparities in CM but extend prior work by demonstrating that these differences persist even in a generation that did not experience the GDR firsthand. Our study suggests that sociopolitical legacies may continue to shape familial environments and risk and protective factors for CM long after reunification. This highlights the need for further investigation into the mechanisms underlying these long-term effects, including potential differences in parenting practices, social norms, and support systems between East and West Germany.

One potential explanation for the lower incidence of physical abuse in East Germany is the existence of former disparate legislative frameworks. Corporal punishment of minors in school was already prohibited in the former German Democratic Republic (GDR) in 1949. In West Germany, corporal punishment in school became prohibited in 1973, and in Bavaria 1983 respectively. This may reflect a greater acceptance of disciplinary strategies involving physical violence in West Germany, which could consequently result in higher prevalence rates of physical abuse [33]. A study on recalled parental disciplinary practices lends support to this assumption. Rejection and punishment as legitimate parenting interventions were reported significantly less often by participants in East Germany compared to West Germans [25]. The assumption that legislation affects physical disciplinary measures is corroborated by the significant decline in physical punishment in Sweden following the prohibition of physical disciplinary measures in 1979 [34]. In Germany, as well, there was a significant decline in physical punishment of children and adolescents following the incorporation of the right to non-violent upbringing into the Civil Code in 2000 [20]. Another potential reason for the observed difference in violent and abusive parenting practices is the greater acceptance of parenting manuals that were widely available during the Nazi era and were also popular in West Germany until the 1980s. Examples of these manuals include Johanna Haarer’s books on child rearing, which were not re-published in the GDR [35].

Our findings regarding sexual abuse in East and West Germany align with those of Schötensack et al. [36]. One potential explanation for the lower prevalence rates in East Germany is the discrepancy in childcare arrangements and the varying status of women in these societies. The provision of full-time childcare in eastern Germany was of greater availability and acceptance than in western Germany, a distinction that persists to the present day, as evidenced by the fact that a greater proportion of women in eastern Germany are employed on a full-time basis [37]. This results in a broader range of support, increased social interaction, and additional reference figures for children. The formation of a broad social network can serve as a protective factor against potential traumatic experiences [38]. Furthermore, the stronger gender hierarchy in West Germany may have rendered children who grew up there more susceptible to sexual violence. Gender inequality is a significant risk factor for sexual violence during childhood [19, 39, 40]. This is largely attributable to the greater financial dependence of women and the concomitant difficulty in leaving an abusive, familial environment.

Furthermore, the GDR distinguished itself within the Soviet Union through its policies on gender equality, universal access to early childcare, and the prohibition of corporal punishment. Unlike many Soviet Union countries, the GDR put an emphasis on state-run childcare facilities, which were not merely supervisory but actively promoted early education and child development. These policies supported gender equality by enabling women to participate fully in the workforce while alleviating familial pressures. Additionally, the GDR formally prohibited corporal punishment earlier and more consistently than other Soviet-aligned states, reflecting a commitment to nonviolent child-rearing practices [41]. It is notably, that these policies were shaped by the GDR’s proximity to and competition with West Germany, as the state sought to present its social policies as superior to those of its capitalist counterpart.

As the results pertain to the post-unification generation, who were too young to have a conscious recollection of the GDR era (only 2.6% of the respondents were older than three years old when the Berlin Wall fell), it is imperative to contextualize the findings within the framework of transgenerational processes. Based on the analyses conducted by Clemens et al. [20] and the classical learning theories (e.g., theories of modeling), it can be assumed that at least some of the parenting experiences are transferred to the children. Brähler and Richter [25, 42] demonstrated significant differences in recalled parenting practices between East and West Germany. The tendency for memories of parenting to be warmer in regions of eastern Germany may be a factor in the transmission of parenting styles across generations. As a case in point, Clemens et al. [20] present the example of the potential transfer of violent parenting, whereby the transmission of violent experiences to children occurs in a cyclical manner. While the majority of parents do not perpetuate this cycle [43], past experiences of violence may contribute to the elevated prevalence rates observed in Western Germany. Future research should explore in greater depth the sociopolitical factors that contribute to the transgenerational transmission of abusive and neglectful parenting behaviors.

Another factor to consider when interpreting our findings is the potential influence of transgenerational mechanisms of denial or fear in reporting childhood maltreatment among the post-unification generation in the former GDR. The historical context of the GDR, with its pervasive state surveillance and a culture of silence, may have contributed to a lasting mistrust of institutions and reluctance to disclose sensitive personal experiences. These societal attitudes could have been unconsciously transmitted across generations, shaping reporting behaviors even among those who did not directly experience the GDR era. As a result, individuals from East Germany may be less likely to acknowledge or disclose experiences of childhood maltreatment, potentially contributing to the observed regional differences in prevalence rates. However, prior studies investigating possible differences in response behavior between East and West Germans have not found systematic differences in the reporting of mental health constructs [44, 45]. This supports the validity of comparing self-reported data between the two groups in our study. Additionally, the items of the CTQ are formulated in a clear and direct manner (e.g., "People in my family hit me so hard that it left me with bruises or marks."), reducing the likelihood of culturally driven response biases. While reporting biases can never be entirely ruled out in retrospective studies, the consistency of our findings with previous research suggests that the observed regional differences are unlikely to be attributable to differential reporting tendencies.

## Limitations

Despite the large sample size and robustness of the effects, several limitations must be acknowledged. Thus, the CTQ is susceptible to memory effects or the concealment of abusive or neglected events due to shame or fear [46]. As a result, there is a possibility of distortion. Additionally, East and West German may differ in their memory, perception and definition of childhood maltreatment. Therefore, there is the possibility that our results are measuring these differences rather than actual occurrences of CM. Furthermore, even though a weighing factor was applied, the data may be subject to longitudinal selection effects, which limit the representativeness of the results. One limitation of this study pertains to the determination of participants’ state of residence, which was based on their current state of residence rather than their childhood residence. Additionally, the absence of information regarding the internal migration status of participants could further distort the results, as individuals who were raised in the new states of Germany may have been erroneously assigned to the old states, and vice versa. This approach may introduce potential confounding effects, particularly due to migration patterns. For instance, individuals who experienced traumatic childhoods in East Germany may have been more likely to migrate to Western states in search of better opportunities or support systems. Such migration could blur the East-West dichotomy by overrepresenting individuals with experiences of CM in Western states. Future studies should address this issue by incorporating data on participants’ childhood residence to better account for the potential impact of migration. One further limitation of the present study is the poor internal consistency of the CTQ subscale for physical neglect, which may affect the reliability of the findings related to this specific form of maltreatment. The low internal consistency of this subscale in our sample could reflect measurement challenges, such as variability in how individuals interpret or report experiences of neglect. Results related to physical neglect should therefore be interpreted with caution.

## Conclusion & implications

Our findings suggest that the four decades of disparate socialization in the formerly divided Germany have left a significant imprint, observable even in subsequent generations, and have influenced the prevalence of abusive and neglected childhood experiences. The social and political context of the GDR appears to have provided a greater degree of protection against the occurrence of abuse and neglect during childhood, a phenomenon that has also manifested in subsequent generations.

The findings of this study have important implications for policy, research, and practice. From a policy perspective, our results support the need to advocate for the prohibition of corporal punishment in regions where it remains legal. Additionally, our results suggest that promoting gender equality and accessible childcare as measures to alleviate parental stress may reduce abusive parental behaviors. Future research should explore the sociopolitical influences on childhood maltreatment in other post-communist states to identify shared and unique factors across different contexts. Additionally, longitudinal studies are needed to investigate the long-term effects of transgenerational parenting practices across varying cultural and political environments. Practically, the findings highlight the importance of implementing community-based parenting programs and accessible mental health services in regions with historically higher abuse prevalence. Training professionals to effectively recognize and address child maltreatment, particularly in areas with sociopolitical vulnerabilities, is also essential for prevention and early intervention. Together, these strategies aim to reduce the prevalence and impact of childhood maltreatment and foster healthier family environments.

## Supplementary Information

Supplementary material 1

## Data Availability

Data and materials are available upon reasonable request from the corresponding author.
