# Supplementary material for: Legacy of the GDR: regional disparities in childhood maltreatment in post-unification Germany
Source: Child Adolesc Psychiatry Ment Health. 2025 Mar 20;19:22. doi: 10.1186/s13034-025-00876-7 (PMC11927179; doi:10.1186/s13034-025-00876-7)
Supplement: Supplementary file 1 — Supplementary material 1 [file 13034_2025_876_MOESM1_ESM.docx]

**Supplement materials**

|  | **Berlin** |  |
| --- | --- | --- |
|  | ***N = 277*** |  |
| **At least one type of Maltreatment** | **N** | ***%*** |
| yes | 37 | 13.6 |
| no | 236 | 86.4 |
|  |  |  |
| **Emotional Abuse** |  |  |
| yes | 13 | 4.7 |
| no | 261 | 95.3 |
|  |  |  |
| **Physical Abuse** |  |  |
| yes | 5 | 1.8 |
| no | 272 | 98.2 |
|  |  |  |
| **Sexual Abuse** |  |  |
| yes | 12 | 4.3 |
| no | 265 | 95.7 |
|  |  |  |
| **Emotional neglect** |  |  |
| yes | 14 | 5.1 |
| no | 263 | 94.9 |
|  |  |  |
| **Physical neglect** |  |  |
| yes | 17 | 6.2 |
| no | 259 | 93.8 |

**Table 4.** Prevalences of abusive and neglectful childhood experiences of the sub-dimensions for Berlin

**Table 5**. Results of the linear regression analysis for the severity of different forms of abuse and neglect

| ***Dependent Variable*** | ***Independent Variable*** | ***Unstand. Beta Coefficient*** | ***Stand. Error for the Beta-Coefficient*** | ***95% confidence interval (CI)*** | ***p*** |
| --- | --- | --- | --- | --- | --- |
|  |  |  |  |  |  |
| **Emotional Abuse** | *Gender* | .625 | .084 | .460 - .790 | **<.001***** |
| (N = 5,669) | *Age* | .038 | .012 | .014 - .063 | **.002**** |
|  | *East-West* | -.465 | .126 | -.713 -(-).218 | **<.001***** |
|  | *High School diploma/ none* | .166 | .088 | -.005 - .338 | .057 |
|  | *Subjective social status* | -1.013 | .087 | -1.184 – (-).842 | **<.001***** |
|  |  |  |  |  |  |
| **Physical Abuse** | *Gender* | .003 | .046 | -.087 - .092 | .950 |
| (N = 5,654) | *Age* | .014 | .007 | .001 - .027 | **.035*** |
|  | *East-West* | -0.208 | 0.068 | -.342 – (-).074 | .002* |
|  | *High School diploma/ none* | -0.125 | 0.047 | -.218 - (-).032 | **.009**** |
|  | *Subjective social status* | -0.255 | 0.047 | -.348 – (-).163 | **<.001***** |
|  |  |  |  |  |  |
| **Sexual Abuse** | *Gender* | .318 | .043 | .233 - .403 | **<.001***** |
| (N = 5,649) | *Age* | .006 | .006 | -.006 - .019 | .330 |
|  | *East-West* | -.160 | .065 | -.287 - (-).032 | **.014*** |
|  | *High School diploma/ none* | -.116 | .045 | -.205 – (-).028 | **.010*** |
|  | *Subjective social status* | -.189 | .045 | -.278 – (-).101 | **<.001***** |
|  |  |  |  |  |  |
| **Emotional Neglect** | *Gender* | -.018 | .105 | -.223 - .187 | .865 |
| (N = 5,667) | *Age* | .104 | .016 | .073 - .134 | **<.001***** |
|  | *East-West* | -.195 | .157 | -.502 - .113 | .215 |
|  | *High School diploma/ none* | -.334 | .109 | -.547 – (-).120 | **.002**** |
|  | *Subjective social status* | -1.262 | .108 | -1.474 – (-)1.049 | **<.001***** |
|  |  |  |  |  |  |
| **Physical Neglect** | *Gender* | -.055 | .059 | -.171 – .060 | .350 |
| (N = 5,670) | *Age* | .071 | .009 | .054 - .089 | **<.001***** |
|  | *East-West* | -.167 | .088 | .545 - .979 | .059 |
|  | *High School diploma/ none* | -.351 | .061 | .550 - .815 | **<.001***** |
|  | *Subjective social status* | -.414 | .061 | .511 - .758 | **<.001***** |

*Notes,* significance levels*:* *** *p* <.001, **  *p* <.01, * *p* <.05
